# Characterization of newly isolated thermotolerant bacterium *Cupriavidus* sp*.* CB15 from composting and its ability to produce polyhydroxyalkanoate from glycerol

**DOI:** 10.1186/s12934-023-02059-5

**Published:** 2023-04-12

**Authors:** Anuyut Yootoum, Kittisak Jantanasakulwong, Pornchai Rachtanapun, Churairat Moukamnerd, Thanongsak Chaiyaso, Chayakorn Pumas, Nuttapol Tanadchangsaeng, Masanori Watanabe, Toshiaki Fukui, Chayatip Insomphun

**Affiliations:** 1 Interdisciplinary Program in Biotechnology, Graduate School, Chiang Mai University, Chiang Mai, 50200 Thailand; 2 School of Agro-Industry, Faculty of Agro-Industry, Chiang Mai University, 155 Mae Hia, Mueang, Chiang Mai, 50100 Thailand; 3 Cluster of Agro Bio-Circular-Green Industry (Agro BCG), Chiang Mai University, Chiang Mai, 50100 Thailand; 4 Department of Biology, Faculty of Science, Chiang Mai University, 239 Huaykaew Road, Suthep, Mueang, Chiang Mai, 50200 Thailand; 5 College of Biomedical Engineering, Rangsit University, 52/347 Lak-Hok, Pathumthani, 12000 Thailand; 6 Graduate School of Agriculture, Yamagata University, 1-23 Wakaba-Machi, Tsuruoka, Yamagata 997-8555 Japan; 7 School of Life Science and Technology, Tokyo Institute of Technology, 4259 Nagatsuta-Cho, Midori-Ku, Yokohama, Kanagawa 226-8503 Japan

**Keywords:** Polyhydroxyalkanoate, Thermotolerant bacterium, *Cupriavidus* sp., Glycerol

## Abstract

**Background:**

This study aimed to isolate a novel thermotolerant bacterium that is capable of synthesizing polyhydroxyalkanoate from glycerol under high temperature conditions.

**Results:**

A newly thermotolerant polyhydroxyalkanoate (PHA) producing bacterium, *Cupriavidus* sp*.* strain CB15, was isolated from corncob compost. The potential ability to synthesize PHA was confirmed by detection of PHA synthase (*phaC*) gene in the genome. This strain could produce poly(3-hydroxybutyrate) [P(3HB)] with 0.95 g/L (PHA content 75.3 wt% of dry cell weight 1.24 g/L) using glycerol as a carbon source. The concentration of PHA was enhanced and optimized based on one-factor-at-a-time (OFAT) experiments and response surface methodology (RSM). The optimum conditions for growth and PHA biosynthesis were 10 g/L glycerol, 0.78 g/L NH_4_Cl, shaking speed at 175 rpm, temperature at 45 °C, and cultivation time at 72 h. Under the optimized conditions, PHA production was enhanced to 2.09 g/L (PHA content of 74.4 wt% and dry cell weight of 2.81 g/L), which is 2.12-fold compared with non-optimized conditions. Nuclear magnetic resonance (NMR) analysis confirmed that the extracted PHA was a homopolyester of 3-hydyoxybutyrate.

**Conclusion:**

*Cupriavidus* sp*.* strain CB15 exhibited potential for cost-effective production of PHA from glycerol.

**Supplementary Information:**

The online version contains supplementary material available at 10.1186/s12934-023-02059-5.

## Background

Due to their fantastic properties and great performance, such as their lightweight, hygienic, and resistant material, petrochemical plastics have played a necessary role in our daily lives. However, few plastics were recycled since they were difficult to be removed and degraded, resulting in large carbon footprints. Some plastics entered into the ocean crack into microplastics that are smaller than 5 mm and contain various chemical additives [1]. These contaminates are extremely harmful to living organisms including humans, and they may be one of the major threats to biodiversity [1, 2].

So far, biodegradable plastics have attracted interest to be a radical eco-innovation by substituting fossil-based sources for sustainable development, because they are derived from renewable biomass resources, as well as they can be completely degraded to CO_2_ and H_2_O under proper conditions. Examples of interesting bioplastics are polylactic acid (PLA), cellulose-based polymers, lignin-based polymers, and polyhydroxyalkanoates (PHAs).

Among these candidates, PHAs are a great family of biodegradable polymers that is a group of natural polyesters. They were accumulated as granules inside microbial cells as carbon and energy storage compounds under excess carbon source and low limiting nutrient (phosphate, nitrogen, and oxygen) conditions by three key enzymes, 3-ketothiolase (PhaA), acetoacetyl-CoA reductase (PhaB), and PHA synthase (PhaC).

In general, PHAs can be classified based on the number of carbon atoms in their carbons units into subclasses: short-chain length PHAs (*scl*-PHAs) consist of 3 to 5 carbon atoms per monomer unit, e.g. poly(3-hydroxybutyrate) [P(3HB)], poly(4-hydroxybutyrate) [P(4HB)], poly(3-hydroxyvalerate) [P(3HV)], and poly(3-hydroxypropionic acid) [P(3HP)] or copolymer such as P(3HB-*co*-4HB) and P(3HB-*co*-3HV) [3, 4] and medium-chain length PHAs (*mcl*-PHAs) with a chain length of 6 to 14 carbon atoms per monomer unit, e.g. poly(3-hydroxyhexanoate) [P(3HHx)], poly(3-hydroxyoctanoate) [P(3HO)], and copolymers such as P(3HHx-*co*-3HO) [5].

Even though PHAs have various applications, the high production costs still obstruct their commercial use. There are many strategies to facilitate economical production of PHA such as utilization of inexpensive and sustainable carbon sources, which are by-products or wastes from agriculture and industries [6]. Additionally, another significant problem for PHA production is the cost of maintaining temperature appropriate for bacterial growth. Certain bacteria become unstable in high-temperature conditions because their process utilizes overheated energy from the bioreactor’s stirring system. Thermotolerant cultivations can be employed as “self-heating systems” because of the heat energy generated by microbial metabolism [7]. In addition, the high-temperature manufacturing of PHAs offers various benefits, including reduced risk of contamination by undesired microorganisms, lower bioreactor cooling costs, and enhanced PHA productivity [8]. Also, cultivation at high temperature provides other advantages, for examples, it enhances the solubility of substrates and reaction rates, reduces the viscosity of the medium, and improves its homogeneity [9]. *Bacillus shackletonii* [10], *Aneurinibacillus thermoaerophilus* [11], *Chelatococcus composti* [12], and *Pseudomonas thermotolerans* [13] are some of the thermotolerant bacteria that have recently been reported to biosynthesize PHAs. Moreover, a variety of thermophilic bacteria are feasible to produce PHAs including *Tepidimonas taiwanensis* [14], *Schlegelella thermodepolymerans* [15], *Aneurinibacillus* spp. [16], and *Cupriavidus cauae* PHS1 [17].

In this research, we isolated novel thermotolerant PHA producing bacteria from agricultural composts. Among them, *Cupriavidus* sp*.* CB15 was the most promising strain that is capable of producing PHA from glycerol as a carbon source. Therefore, this strain was investigated the best conditions for highest PHA productivity by statical design experiment.

## Materials and methods

### Isolation and selection of the thermotolerant bacteria accumulating polyhydroxyalkanoates

Four different agricultural composts such as corncob, coconut coir, coffee pulp and leaves compost were collected from Mae Hia Agricultural Research, Demonstrative and Training Center (18° 45′38.5″N: 98° 56′10.4″E) Chiang Mai, Thailand. Then, agricultural composts were then serially diluted and spread on a nitrogen-limited mineral salts (MB) agar plate medium [18] consisting of 9.0 g of Na_2_HPO_4·_12H_2_O, 1.5 g of KH_2_PO_4_, 0.5 g of NH_4_Cl, 0.2 g of MgSO_4·_7H_2_O, 10 g of glycerol and 15 g of agar per liter of distilled water. The medium was autoclaved at 121 °C and 103.4 kPa for 15 min. After cooling, 1 mL/L of trace element solution [19] and 0.5 mg/mL Nile red (Sigma, India) in dimethyl sulfoxide (DMSO) were added. The colonies were observed after 3–4 days at 45 °C (depending on composting samples). Positive single colonies that showed bright orange fluorescence under UV light were selected and streaked on the MB agar plate medium. The bacterial strains were maintained in 25% glycerol at − 20 °C for further use.

### Determination of the growth conditions

A single colony picked from the MB plate media was transferred into a 15 mL bottle containing 5 mL of the nutrient medium at 45 °C overnight. Then, 1% (v/v) of the fresh seeds was inoculated into a 250 mL Erlenmeyer flask with 100 mL of MB medium containing 1% (w/v) glycerol as a carbon source and incubated at 45 °C with constant shaking of 200 rpm for 72 h. The cells were collected by centrifugation (7871 ×*g*, 4 °C for 10 min). The cells were washed twice with 70% (v/v) ethanol and distilled water, respectively. After that, the cells were lyophilized for calculation of dry cell weight (DCW, g/L). All experiments were done in triplicate.

### Quantitative analysis of PHA

PHA within the lyophilized cells was subjected to methanolysis [19]. Two milliliters of acidified methanol solution (15% H_2_SO_4_ (v/v)) and 2 mL chloroform were added to approximately 25 mg of lyophilized cells in a 15 mL screw cap glass tube. Then, the reaction mixture was incubated on a digital dry bath at 100 °C for 140 min. After cooling at ambient temperature, 1 mL of distilled water was added. The organic layer was used for gas chromatography (GC) analysis on a 7890B GC system (Agilent, UK) equipped with a HP-5 column (30 m × 0.25 mm × 0.25 µm) (Agilent, USA) and a flame ionization detector. Helium was a carrier gas with a flow rate of 0.8 mL/min. The injector and detector temperature were 280 °C. The initial oven temperature was 100 °C (maintained for 1 min) and increased to 280 °C with a rate of 8 °C/min (maintained for 10 min). 1 µL of each sample was introduced into the inlet with split ratio 20:1. The internal standard was methyl octanoate (Sigma-Aldrich, Germany) and P(3HB) (Sigma-Aldrich, USA) was used as the external standard.

### Identification and biochemical characterization of the selected strain

Genomic DNA was extracted and purified using PureDirex genomic DNA isolation kit (Bio-helix, Taiwan) and was used for PCR amplification. 16S rRNA gene was amplified by KOD One™ PCR master mix (Toyobo, Japan) using the forward primer 27F (5′-AGA GTT TGA TCM TGG CTC AG-3′) and the reverse primer 1492R (5′-TAC GGY TAC TTG TTA CGA CTT-3′). The PCR reaction was performed in a thermal cycler (Analytikjena, Germany). The amplified PCR products were purified according to the manufacturer’s protocol (Bio-helix, Taiwan). 16S DNA sequencing was conducted by ATCG Co., Ltd. (Pathumthani, Thailand). The nucleotide sequence was aligned with the related sequences in NCBI database. Phylogenetic tree was constructed using Molecular Evolutionary Genetics Analysis (MEGA, version 7.0.26) software with the Neighbor-joining method.

The genomic DNA of the CB15 strain was used as a template for PCR amplification of PHA synthase (*phaC*) with forward primer G-D (5′-GTG CCG CCS YRS ATC AAC AAG T-3′) and reverse primer G2-R (5′-GTA GTT CCA SAY CAG GTC GTT-3′) [20].

IMViC-test (Himedia, India), citrate test, and catalase test were performed. The ability to grow on various sugars such as glucose, xylose, arabinose, sucrose, and fructose were conducted by inoculating the bacterium into Phenol Red Broth Base (PRBB) medium (Himedia, India) containing 1% (w/v) of each sugar.

### Optimization of nutritional and physical parameters for efficient PHA production by one factor at a time (OFAT)

The isolate showing the highest PHA production was selected for optimization of nutritional and physical parameters on PHA biosynthesis. The effect of age of inoculum was investigated by using inoculum grown for 6, 9, 12, 15, 18, and 21 h. The effect of glycerol concentration (1%, 2%, 3%, 4%, 5%, and 6% (w/v)) was observed. The effect of different nitrogen sources was studied by adding different nitrogen sources (NH_4_Cl, (NH_4_)_2_SO_4_, (NH_4_)_2_HPO_4_, urea, and yeast extract) with the best glycerol concentration. Finally, the effect of nitrogen concentration was studied by adding the best nitrogen source at concentration between 0 and 3 g/L with the best glycerol concentration. Each condition was incubated at 45 °C with shaking speed of 200 rpm for 72 h using the best age of inoculum selected.

After optimization of nutritional parameters, effects of physical conditions such as initial pH and shaking speed were studied. The initial pH of the medium was prepared at 5.0, 6.0, 7.0, 8.0, 9.0, and without adjusting pH (approximately 7.3). The isolate was cultured in MB medium containing the best carbon and nitrogen sources at the optimum concentration and incubated at 45 °C and 200 rpm shaking for 72 h. To determine the effect of shaking speed, the medium was prepared by using the carbon source, nitrogen source, and pH that produced the highest PHA and incubated at 45 °C with different shaking speed at 100, 125, 150, 175, 200, and 225 rpm for 72 h. DCW (g/L), PHA production (g/L), and PHA content (wt%) were investigated in all the experiments, which were done in triplicates.

### Process optimization for efficient PHA production using response surface methodology (RSM)

The response surface methodology (RSM) using a set of central composite designs (CCD) were applied to determine the optimum condition and clarify the interaction between the significant variables that influence the growth and PHA production based on OFAT. Each independent variable: NH_4_Cl concentrations (X_1,_ g/L), temperature (X_2_, °C), and shaking speed (X_3,_ rpm) was studied at five different levels (-α, -1, 0, + 1, + α). Sixteen experiments constructed by Design Expert (version 11.0.1) was employed. The study was performed in 250 mL Erlenmeyer flasks containing 100 mL MB medium with age of inoculum at 9 h (OD_600_ = 2.321) and 1% (w/w) glycerol. The following quadratic model was used for regression analysis:

$$y={a}_{0}+\sum_{i=1}^{n}{a}_{i}{x}_{i}+{\sum }_{i=1}^{n}\sum_{j=1+1}^{n}{a}_{ij}{x}_{i}{x}_{j}+\sum_{i=1}^{n}{a}_{ii}{x}_{i}^{2},$$

where Y is predicted response (DCW, PHA production); ${a}_{0}$ is constant; ${a}_{i,}{a}_{ii,}{a}_{ij}$ are regression coefficients for individual, square, and interaction terms; ${x}_{i},{x}_{j}$ are factors; i, j = 1, 2, 3

DCW (g/L) and PHA production (g/L) were taken as response 1 and response 2 of the dependent factors, respectively. The statistical parameters were determined by using analysis of variance (ANOVA). The significance of the model equation and model terms were evaluated by the Fisher’s *F*-test. The quality of the fit of polynomial equation was expressed by the coefficient of determination (*R*^2^). The fitted polynomial equation was expressed as 3D surface plots to express the relationship between the responses and the experimental levels of each variable.

### Statistical analysis

All experiments were conducted in triplicate (n = 3) and the results were represented as means ± standard deviations (SD). All the statistical data were carried out in Graph Pad Prism version 8.4.2.

### Growth curve/production kinetics under optimal medium and conditions

These experiments were undertaken in a 250 mL Erlenmeyer flask with 100 mL working volume to investigate the fermentation course for the PHA production under optimal medium and conditions at 45 °C for 96 h. The cells were collected from each flask every 12 h. DCW (g/L), PHA production (g/L), and PHA content (wt%) were investigated. Glycerol concentration in the medium solution was measured using a HPLC Chromaster system equipped with a RI detector (Hitachi, Japan) and an Aminex HPX-87H column (BIO-RAD, USA). The column temperature was maintained at 40 °C and eluted with 5 mM H_2_SO_4_ at a flow rate of 0.75 mL/min. Ammonium nitrogen concentration was determined at 425 nm by Nessler’s reagent method using HACH DR/2010 spectrophotometer (Hach, USA). The experiment was carried out in triplicate.

### Polymer extraction

The polymer was extracted from the bacterial cells as described before with slight modifications [21]. Briefly, the lyophilized cells were stirred in chloroform (ACI LABSCAN, Thailand) for 60 h at room temperature. Cell debris was removed by filtration. The filtrate was purified by precipitation with tenfold volume of methanol (Honeywell, USA). The purified polymer was dried in a desiccator before weighing.

### Nuclear magnetic resonance (NMR) spectroscopy analysis

The chemical compositions of the polymer were determined by ^1^H NMR spectra (Bruker Avance NEO 500 MHz spectrometer, Bruker, Germany). The 500 MHz ^1^H NMR spectra were recorded in CDCl_3_ solution (Sigma-Aldrich, USA) of 20 mg/mL PHA at 25 °C.

## Results and discussion

### Primary isolation of thermotolerant PHA-producing bacteria

This study focused on the isolation of thermotolerant PHA-producing bacteria from agricultural composts in Chiang Mai, Thailand. The properties of compost showed a potential environment for the isolation of wild-type PHA accumulating bacteria (Additional file 1: Table S1). Out of the fifty isolates, 18 isolates emitted orange fluorescence of Nile red under UV light, suggesting that they were capable of accumulating PHA within the cells. Each the fluorescent isolate was picked and sub-cultured on MB agar medium containing glycerol. These isolates were named according to types of composts-leave compost (AP-01, 05, 06), corncob compost 1 (CB-11, 12, 13, 14, 15, 16, 18, 19), corncob compost 2 (CB-20, 21, 27, 28), and coffee pulp compost (GP-03, 07, 09).

### Quantitative analysis of PHA production

**Fig. 1:**
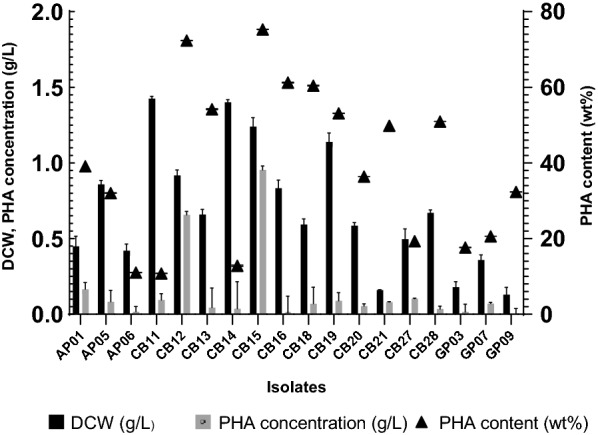
PHA production by isolate strains. Each isolate was cultivated in a mineral salt medium containing 1% (w/v) glycerol at 45 °C for 72 h

The ability of the isolates in PHA production was investigated. The 18 isolates were cultured in 100 mL MB medium containing 1% (w/v) glycerol as a carbon source and incubated at 45 °C and 200 rpm for 72 h. The results showed that all the isolates could produce PHA (Fig. 1). Among the isolates, the isolate CB15 produced PHA with the highest concentration of 0.95 g/L (PHA content 75.3 wt%). Therefore, the isolate CB15 was selected for further studies.

### Morphological and molecular characterization of the isolate CB15

**Fig. 2:**
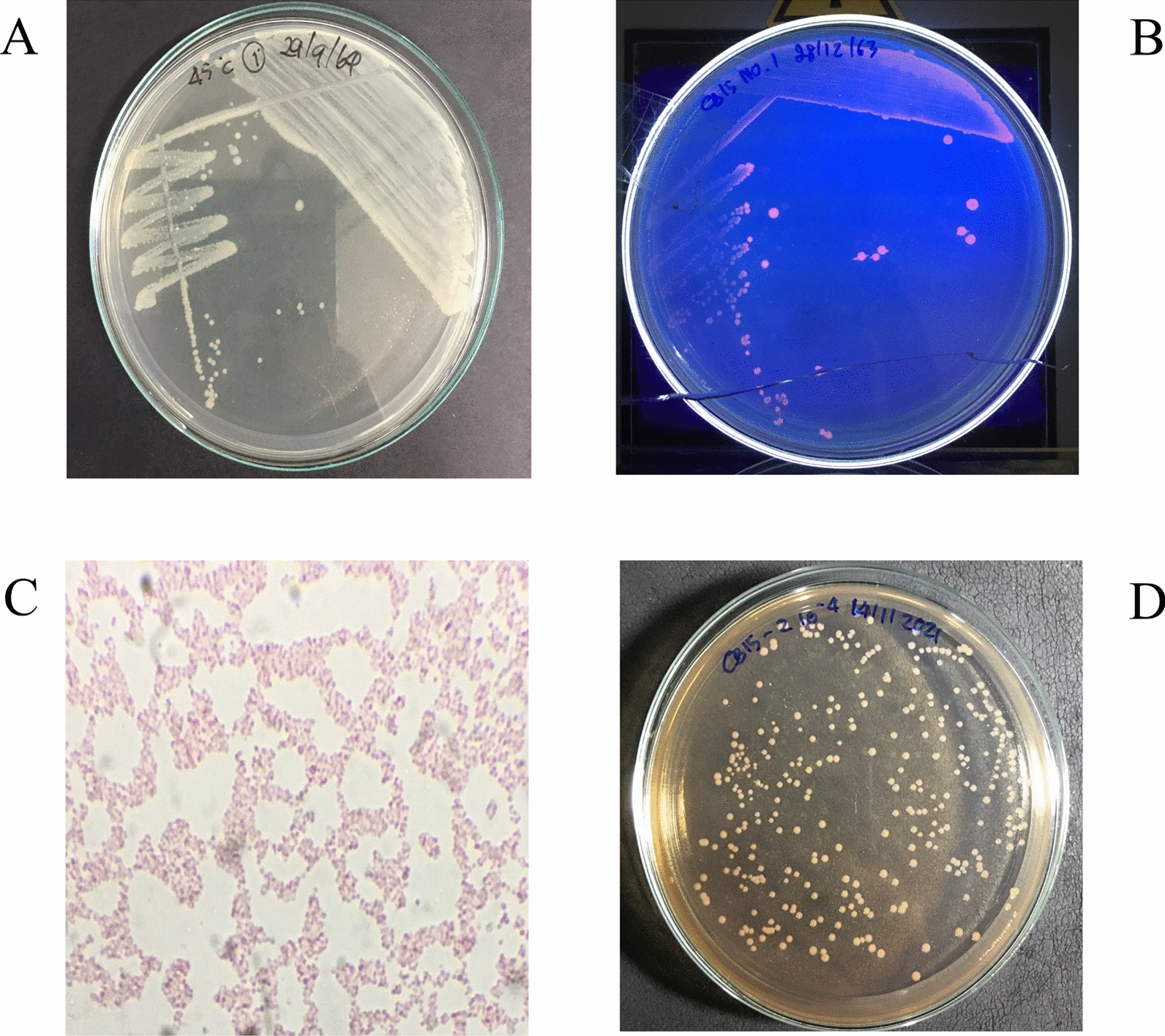
Morphology of *Cupriavidus* sp. CB15. **A** Morphology of the CB15 on Nutrient agar, **B** Nile Red staining under UV light, **C** Gram staining of the CB15 at 100× magnification, and **D** Morphology of the CB15 on MacConkey agar

Colonies of CB15 on nutrient agar medium were small, circular with entire margins, white-colored and mucoid (Fig. 2A). While growing on MB agar supplemented with Nile red, the isolated CB15 showed strong orange fluorescence under UV light irradiation (Fig. 2B). The bacterium CB15 was a gram-negative rod-shaped, facultative anaerobe and non-spore-forming (Fig. 2C). It was able to grow on MacConkey agar medium, but it could not ferment lactose (Fig. 2D). The biochemical characteristics of the strain CB15 were examined (Table 1). It showed positive results on citrate and catalase tests, whereas negative results on indole, methyl red and Voges–Proskauer tests. The isolate could assimilate glycerol and urea. Nevertheless, CB15 could not utilize the sugars tested in this study.

**Table 1:** Biochemical characteristics of CB15 isolated strain

Characteristics	*Cupriavidus* sp. CB15	*Cupriavidus* *gilardii* LMG 5886 [22, 23]	*Cupriavidus* *taiwanensis* LMG 19424 [22, 23]	*Cupriavidus necator* sp. [22, 23]	*Cupriavidus plantarum* sp. [22, 23]
Indole test	−	−	+	−	−
Methyl red test	−	−	−	−	−
Voges–Proskauer test	−	−	−	−	−
Citrate test	+	−	+	+	−
Catalase test	+	+	+	+	+
Growth at 42 °C	+	+	+	−	+
Assimilation of					
d-Glucose	−	−	−	+	−
d-Xylose	−	−	+	−	−
d-Arabinose	−	−	+	−	−
d-Fructose	−	−	−	+	−
d-Lactose	−	−	−	−	−
Sucrose	−	+	−	+	−
Glycerol	+	+	−	+	−
Urea	+	−	−	+	+

(+) Positive response and (−) negative response

**Fig. 3:**
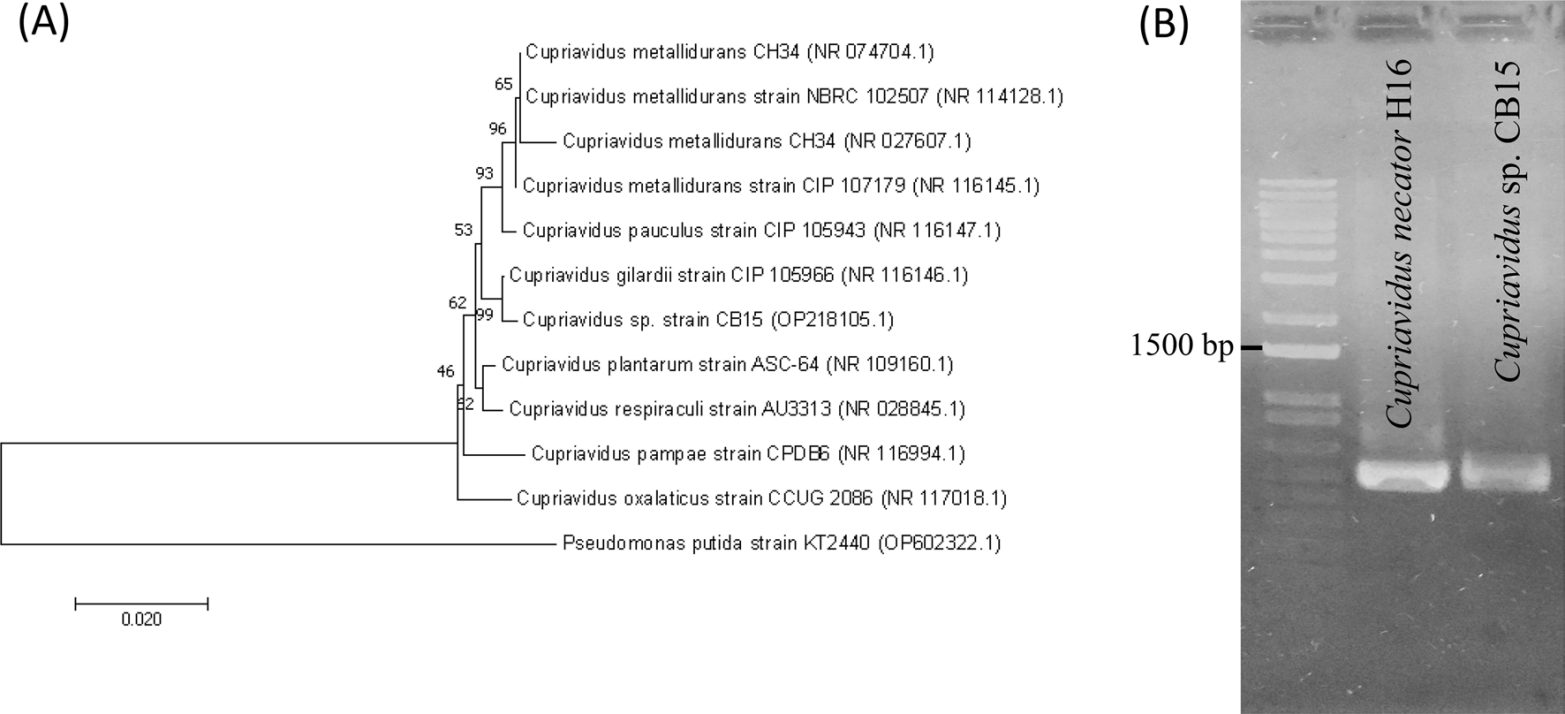
**A** Phylogenetic tree of the CB15 based on partial 16S rDNA sequences. **B** PCR amplification of *phaC* gene using G-D and G2-R primers. Genomic DNA of *Cupriavidus necator* H16 was used as a positive control. The tree was constructed by the neighbor-joining method in the bootstrap test (1000 replicates). Bar = 20% sequence divergence. *Pseudomonas putida* strain KT2440 was taken as outgroup for tree formation

To identify the isolate, 16S rRNA analysis was conducted. The partial sequence of the 16S rDNA gene (1402 bp) was deposited to the GenBank database with the accession number OP218105.1. The phylogenetic analysis revealed that this strain is a member of the genus *Cupriavidus* (Fig. 3A). The 16S rDNA sequence of CB15 were 99% identical to *Cupriavidus gillardii* (NR_116146.1). *Cupriavidus neactor* (formerly known as *Ralstonia eutropha*), a member of *Cuprividus* species, have been reported to be one of the most efficient producers of scl-PHAs from a variety of carbon sources [24–26], while there is no information about PHA biosynthesis by *C. gillardii* to date. The CB15 strain and *C. gillardii* differed in their nutritional characteristics that CB15 utilized citrate and urea, which was not utilized by *C. gillardii*, whereas CB15 could not utilize sucrose, which was utilized by *C. gillardii* (Table 1). The strain CB15 was thus named as *Cupriavidus* sp. CB15 based on these results.

The presence of PHA synthase gene (*phaC*) in the CB15 genomic DNA was investigated by PCR using general primers for partial amplification of the gene encoding type I and II PHA synthases [20]. The successful amplification of approximately 540 bp length confirmed the presence of *phaC* (Fig. 3B).

### Optimization for PHA production by one factor at a time approach

#### Inoculum age

**Fig. 4:**
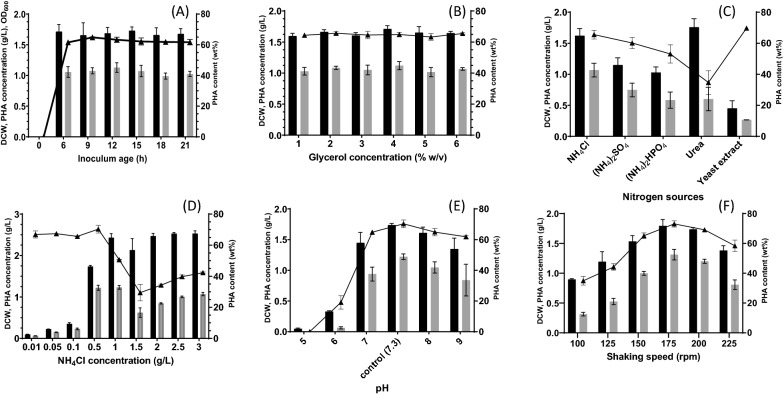
Optimization of PHA production by *Cupriavidus* sp. CB15 under submerged fermentation. CB15 was cultured in mineral salt medium containing 1% (w/v) glycerol at 45 °C for 72 h with shaking at 200 rpm. **A** Inoculum age, **B** Glycerol concentration, **C** Nitrogen sources, **D** NH_4_Cl concentration, **E** pH, and **F** Shaking speed. Dry cell weight (filled with dark bar), PHA production (filled with gray bar), and PHA content (wt%) (dark triangle up) were examined. Data shown are the means and standard deviations of triplicate experiments

PHA production was carried out with an inoculum with different age. As shown in Fig. 4A, inoculum age did not significantly affect to growth and PHA accumulation. The maximum PHA content of 64.8 wt% (PHA 1.08 g/L) was achieved with 1% (v/v) inoculum of the 9-h old cells. The PHA content was slightly decreased when inoculum age was older than 9 h. Ramadas et al. also reported that inoculum age was not a significant parameter for PHA production and biomass [27]. Then, the inoculum age of 9 h was selected for further experiments.

#### Glycerol concentration

The strain CB15 had a potential to produce a high amount of PHA using glycerol as a sole carbon source as describe above. To maximize PHA biosynthesis, different glycerol concentrations (1–6% w/v) were applied to MB medium. PHA concentration (1.02–1.12 g/L) and DCW (1.60–1.71 g/L) were comparable at all tested glycerol concentration (Fig. 4B). This result indicated that glycerol was a favorable substrate for cell growth and PHA accumulation of CB15. Gahlawat and Soni have also mentioned that different concentration of glycerol (10–60 g/L) did not significantly affected the growth rate of *C. necator* [28]. From the result, 1.0% (w/v) glycerol was selected for further experiment.

#### Nitrogen sources and nitrogen concentration

Nitrogen source plays a major role in both cell growth and PHA production [29]. In this study, different inorganic nitrogen sources (NH_4_Cl, (NH_4_)_2_SO_4_, and (NH_4_)_2_HPO_4_) and organic nitrogen sources (urea and yeast extract) were investigated for PHA production. The highest PHA accumulation was observed by NH_4_Cl (1.07 g/L), followed by (NH_4_)_2_SO_4_ (0.75 g/L) and (NH_4_)_2_HPO_4_) (0.58 g/L) (Fig. 4C), where the organic nitrogen sources mainly support cell growth rather than PHA biosynthesis [30]. The highest biomass (1.76 g/L) was obtained in the presence of urea, but the concentration of PHA was not significant (0.60 g/L). Whereas, yeast extract did not support cell growth and PHA production, indicating that CB15 could not utilize complex nitrogen sources. NH_4_Cl has been reported to be the most suitable nitrogen source for PHA accumulation in *C. necator* [24], *C. taiwanensis* [31], *Bacillus subtilis* NG05 [32], *Haloferax mediterranei* [33], and *Vibrio azureus* BTKB33 [34, 35] probably due to its simple structure that can metabolize rapidly.

The nitrogen concentration highly impacted on PHA production. The highest PHA concentration of 1.22 g/L with DCW 1.74 g/L was obtained in the presence of 0.5 g/L NH_4_Cl. When CB15 was cultivated in MB medium containing more NH_4_Cl with higher than 0.5 g/L, biomass was significantly increased, but PHA accumulation was inhibited. This result indicated that the CB15 strain produced PHA under nitrogen-limiting condition. Previous studies also reported that nitrogen limitation was necessary to induce PHA production [36–39].

#### Initial pH value

Effect of initial pH value on PHA production was studied by adjusting the pH of MB medium at 5.0, 6.0, 7.0, 8.0, and 9.0, and without adjusting pH (approximately 7.3). The result indicated that pH range of 7–9 was favorable for PHA production with *Cupriavidus* sp. CB15. The maximum DCW (1.74 g/L) and PHA concentration (1.22 g/L) was obtained with the non-adjusting pH medium (pH 7.3) (Fig. 4E). Similar observation has been previously reported in *Bacillus mycoides* DFC1 [40], while initial pH of 7 was optimal for P(3HB) production in *C. necator* [41], *C. necator* TISR1095 [42], and *C. taiwanensis* 184 [31]. Many studies reported that bacteria could produce PHAs in pH range of 6.0–9.0 [32, 35, 43, 44]. Cell growth (0.33 g/L) and PHA production (0.06 g/L) were drastically decreased at pH 6.0. No PHA production was observed at pH 5.0. Initial pH of culture medium is an important factor for bacterial growth and biosynthesis of metabolites. It has been reported that pH condition higher and lower than the optimum point affected PHA metabolism [45]. PHA accumulation was inhibited at higher pH values, whereas PHA utilization was completely suppressed [46].

#### Shaking speed

The influence of shaking speed on PHA production was studied by varying shaking speed from 100 to 225 rpm. DCW and PHA production were observed to be increased with elevated shaking speed. The maximum DCW (1.79 g/L) and PHA production (1.31 g/L) were observed at shaking speed at 175 rpm (Fig. 4F). Cell growth and PHA concentration were decreased when shaking speed was greater than 175 rpm. Tripathi et al. have reported that shaking speed showed significant influence on P(3HB) accumulation in *Cupriavidus* sp. [47]. Shaking is an important parameter for oxygen and mass transfers. Lower shaking speed may cause cell aggregation, making the medium more heterogenous. Eventually, cell growth ceases and affects PHA production. On the contrary, shaking speed higher than the optimum level may reduce PHA formation due to high oxygen availability. PHA is produced and stored as granules in a cytoplasm when bacteria are under stress conditions, i.e., limitation of nutrient or electron acceptor such as oxygen [48]. Under oxygen limitation condition, the intracellular NADH/NAD^+^ ratio increases resulting in inhibition of citrate synthase and isocitrate dehydrogenase in tricarboxylic acid (TCA) cycle. Therefore, carbon flow is directed toward PHA biosynthesis by converting acetyl-CoA to acetoacetyl-CoA [49].

### Optimization of PHA production using CCD models

Based on the preliminary results, CCD was used to estimate optimum NH_4_Cl concentration, temperature, and shaking speed to maximize cell growth and PHA production. A total of 16 experiments and results are represented in Table 2. The results indicated that the actual responses for PHA production and DCW were correlated with the predicted responses. The highest PHA production at 2.09 g/L with 2.81 g/L of DCW was obtained in standard order no. 16 using the medium containing 0.78 g/L NH_4_Cl, cultivation at 45 °C, and shaking at 175 rpm, which was increased 2.12-fold over the unoptimized condition. Regression equations for each response variable obtained from RSM are as follows:

$${\mathrm{Y}}_{1}(\mathrm{g}/\mathrm{L})=2.72+0.6482{\mathrm{X}}_{1}-0.5905{\mathrm{X}}_{2}+0.0417{\mathrm{X}}_{3}-0.5013{\mathrm{X}}_{1}{\mathrm{X}}_{2}+0.1146{\mathrm{X}}_{1}{\mathrm{X}}_{3}-0.1778{\mathrm{X}}_{2}{\mathrm{X}}_{3}-0.5476{\mathrm{X}}_{1}^{2}-0.6316{\mathrm{X}}_{2}^{2}-0.6045{\mathrm{X}}_{3}^{2}$$

$${\mathrm{Y}}_{2}(\mathrm{g}/\mathrm{L})=1.98+0.2942{\mathrm{X}}_{1}-0.3183{\mathrm{X}}_{2}-0.0469{\mathrm{X}}_{3}-0.2140{\mathrm{X}}_{1}{\mathrm{X}}_{2}+0.0178{\mathrm{X}}_{1}{\mathrm{X}}_{3}-0.0554{\mathrm{X}}_{2}{\mathrm{X}}_{3}-0.4936{\mathrm{X}}_{1}^{2}-0.4904{\mathrm{X}}_{2}^{2}-0.6203{\mathrm{X}}_{3}^{2}$$

where Y_1_ is the predicted response for DCW, Y_2_ is the predicted response for PHA production, and X_1_, X_2_ and X_3_ are the codes terms of the three variables: NH_4_Cl concentration (X_1_), temperature (X_2_), and shaking speed (X_3_), respectively.

**Table 2:** Central Composite Design along with experimental and predicted responses for PHA production by *Cupriavidus* sp. CB15

Standard order	Factors			Response 1	Predicted Response 1	Response 2	Predicted Response 2	Response 3	Predicted Response 3
	NH_4_Cl (g/L)	Temperature (°C)	Shaking speed (rpm)	DCW (g/L)	DCW (g/L)	PHA production (g/L)	PHA production (g/L)	PHA content (wt%)	PHA content (wt%)
1	0.05	40.00	125.00	0.22	0.27	0.15	0.19	68.0	53.0
2	1.50	40.00	125.00	2.06	2.34	0.93	1.17	45.5	53.7
3	0.05	50.00	125.00	0.41	0.45	0.09	0.10	22.1	16.4
4	1.50	50.00	125.00	0.37	0.51	0.15	0.22	40.3	36.0
5	0.05	40.00	225.00	0.40	0.48	0.15	0.17	37.4	35.6
6	1.50	40.00	225.00	2.83	3.01	1.14	1.23	40.2	39.9
7	0.05	50.00	225.00	0.01	− 0.05	0	− 0.14	0	− 14.3
8	1.50	50.00	225.00	0.30	0.47	0	0.05	0	9.0
9	0	45.00	175.00	0.03	0.08	0	0.09	0	19.1
10	1.99	45.00	175.00	2.61	2.26	1.30	1.08	49.7	39.2
11	0.78	36.59	175.00	2.17	1.93	1.32	1.13	60.6	63.0
12	0.78	53.41	175.00	0	− 0.06	0	0.06	0	6.2
13	0.78	45.00	90.91	1.14	0.94	0.47	0.30	41.4	48.4
14	0.78	45.00	259.09	1.19	1.08	0.11	0.15	9.5	11.0
15	0.78	45.00	175.00	2.57	2.72	1.84	1.98	71.6	72.3
**16**	**0.78**	**45.00**	**175.00**	**2.81**	**2.72**	**2.09**	**1.98**	**74.4**	**72.3**

Bold values indicate the best result from 16 experiments

The significance of the model for DCW and PHA production was evaluated by analysis of variance (ANOVA) (Tables 3 and 4). The ANOVA results indicated that the individual terms (X_1_ and X_2_), quadratic terms (X_1_^2^, X_2_^2^ and X_3_^2^), and product term X_1_X_2_ were significant model terms (*p* < 0.05) for both DCW and PHA production.

**Table 3:** ANOVA results based on the regression model for Dry cell weight

Source	Sum of squares	df	Mean Square	F-value	p-value	
Model	18.29	9	2.03	26.82	0.0004	Significant
X_1_-NH_4_Cl	5.74	1	5.74	75.73	0.0001	
X_2_-Temperature	4.76	1	4.76	62.86	0.0002	
X_3_-Shaking speed	0.0237	1	0.0237	0.3133	0.5959	
X_1_X_2_	2.01	1	2.01	26.53	0.0021	
X_1_X_3_	0.1051	1	0.1051	1.39	0.2835	
X_2_X_3_	0.2530	1	0.2530	3.34	0.1174	
X_1_^2^	2.78	1	2.78	36.67	0.0009	
X_2_^2^	3.70	1	3.70	48.79	0.0004	
X_3_^2^	3.39	1	3.39	44.68	0.0005	
Residual	0.4546	6	0.0758			
Lack of fit	0.4258	5	0.0852	2.96	0.4139	Not significant
Pure error	0.0288	1	0.0288			
Cor total	18.74	15				
SD	0.2753					
R^2^	0.9757					
Adjusted R^2^	0.9394					
Predicted R^2^	0.8211					
Adeq precision	14.1085

**Table 4:** ANOVA results based on the regression model for PHA production

Source	Sum of squares	df	Mean square	F-value	p-value	
Model	7.48	9	0.8311	19.51	0.0009	Significant
X_1_-NH_4_Cl	1.18	1	1.18	27.75	0.0019	
X_2_-Temperature	1.38	1	1.38	32.48	0.0013	
X_3_-Shaking speed	0.0300	1	0.0300	0.7052	0.4332	
X_1_X_2_	0.3663	1	0.3663	8.60	0.0262	
X_1_X_3_	0.0025	1	0.0025	0.0596	0.8153	
X_2_X_3_	0.0246	1	0.0246	0.5771	0.4762	
X_1_^2^	2.26	1	2.26	53.00	0.0003	
X_2_^2^	2.23	1	2.23	52.32	0.0004	
X_3_^2^	3.56	1	3.56	83.69	< 0.0001	
Residual	0.2556	6	0.0426			
Lack of fit	0.2241	5	0.0448	1.43	0.5595	Not significant
Pure error	0.0314	1	0.0314			
Cor total	7.74	15				
SD	0.2064					
R^2^	0.9670					
Adjusted R^2^	0.9174					
Predicted R^2^	0.7621					
Adeq precision	13.0121					

The model F-value for DCW and PHA production were 26.82 and 19.51, respectively. Lack of fit value was not significant relative to pure error for both DCW and PHA production, which indicated that the models were precise to predict the response variables. The fit of model was also expressed by the coefficient of determination (R^2^) values, which were 0.9757 for DCW and 0.9670 for PHA production. This suggested that both models could explain more than 96% variable in DCW and PHA production. The predicted R^2^ was 0.8211 for DCW and 0.7621 for PHA production, which was in agreement with the adjusted R^2^ of DCW (0.9394) and PHA production (0.9174).

**Fig. 5:**
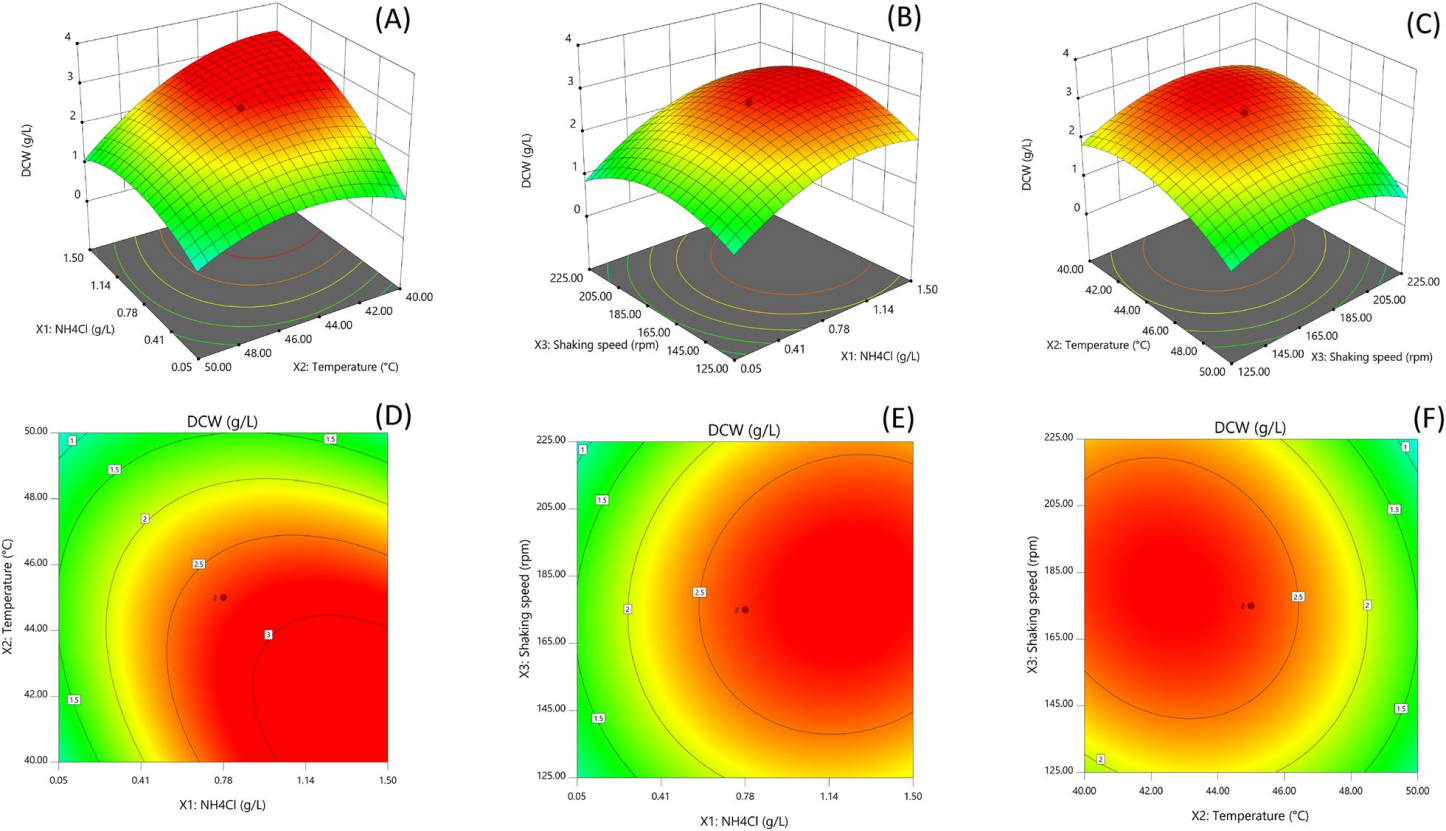
3D surface and contour plots showing the interaction effect of **A**, **D** NH_4_Cl concentration and temperature; **B**, **E** NH_4_Cl concentration and shaking speed; **C**, **F** Temperature and shaking speed on DCW by *Cupriavidus* sp. CB15

**Fig. 6:**
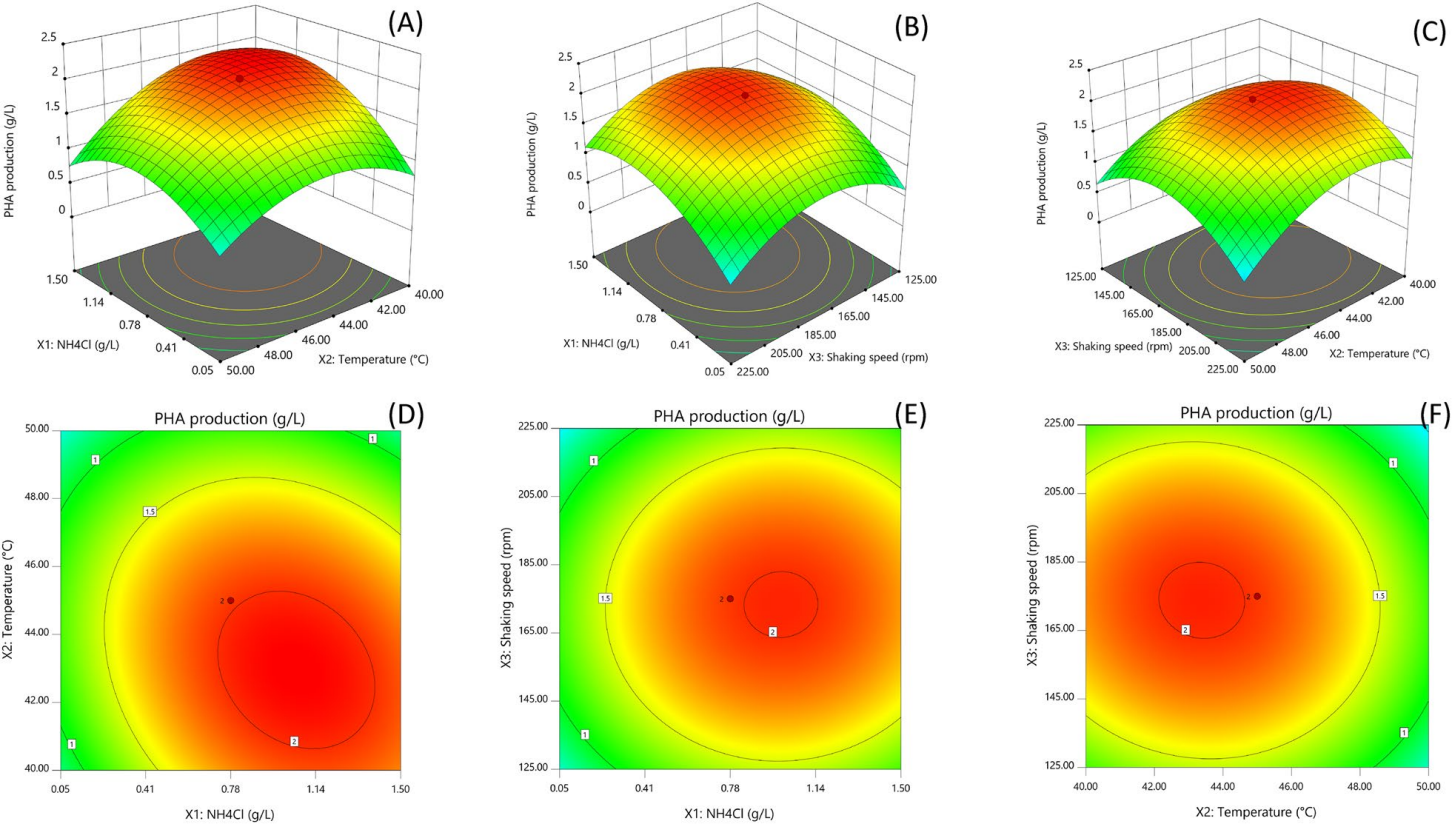
3D surface and contour plots showing the interaction effect of **A**, **D** NH_4_Cl concentration and temperature; **B**, **E** NH_4_Cl concentration and shaking speed; **C**, **F** Temperature and shaking speed on PHA production by *Cupriavidus* sp. CB15

To determine the optimal levels of NH_4_Cl concentration, temperature, and shaking speed, 3D surface and contour plots were constructed by plotting the individual responses (DCW and PHA production) against two selected variables within the experimental range while the other variable was kept at a constant level (Figs. 5 and 6). NH_4_Cl concentration and temperature showed significant positive effect for DCW (Fig. 5A and D). While, interactive effect of NH_4_Cl concentration and shaking speed (Fig. 5B and E) as well as temperature and shaking speed (Fig. 5C and F) was negligible. For PHA production, interactive effect of NH_4_Cl concentration and temperature (Fig. 6A and D) was the most significant, whereas other interactive effects were not significant (Fig. 6B, C, E and F).

### Time course study on PHA production

**Fig. 7:**
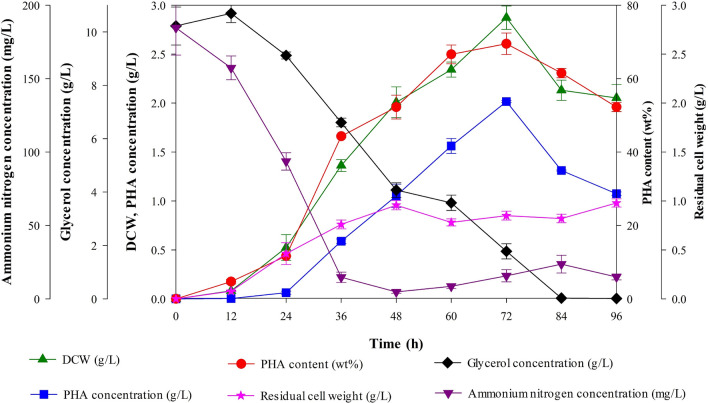
Effect of cultivation time on cell growth and PHA production. *Cupriavidus* sp. CB15 was cultivated in a 100 mL Erlenmeyer flask under optimal conditions. Dry cell weight (green triangle up), PHA concentration (blue square), PHA content (wt%) (red circle), residual cell weight (g/L) (pink star), glycerol concentration (g/L) (black diamond), and ammonium nitrogen concentration (mg/L) (purple triangle down) were examined. Data shown are the means and standard deviations of triplicate experiments

*Cupriavidus* sp. CB15 was cultivated under the optimal conditions, which was 1.0% (w/v) glycerol, 0.78 g/L NH_4_Cl, incubation at 45 °C, and shaking speed at 175 rpm. The time profile for cell growth, PHA production, and utilization of glycerol and ammonium nitrogen are shown in Fig. 7. PHA production was slightly increased when the culture reached log phase (12–24 h) with the decrease in both of glycerol and ammonium nitrogen. After 36 h, PHA concentration was significantly increased due to the depletion of nitrogen source. The maximum PHA concentration (2.01 g/L) was obtained at 72 h cultivation. After 72 h, DCW and PHA concentration were drastically decreased due to the depletion of glycerol (84 h), while residual cell weight remained constant indicating that the storage PHA was mobilized for cell maintenance. This indicated that PHA biosynthesis in this bacterium was partially growth-associated. This result was consistent with partly growth-associated PHA producing strains, for examples, *C. necator* ATCC 17,697 [50], *Cupriavidus* sp. KKU38 [51], and *C. necator* (CCUG52238T) [52] that the growth associated PHA production was observed at the early stage of cultivation with a nitrogen-sufficient condition and PHA accumulation is enhanced by limitation of nitrogen source with excess of carbon source.

### Characterization of PHA

**Fig. 8:**
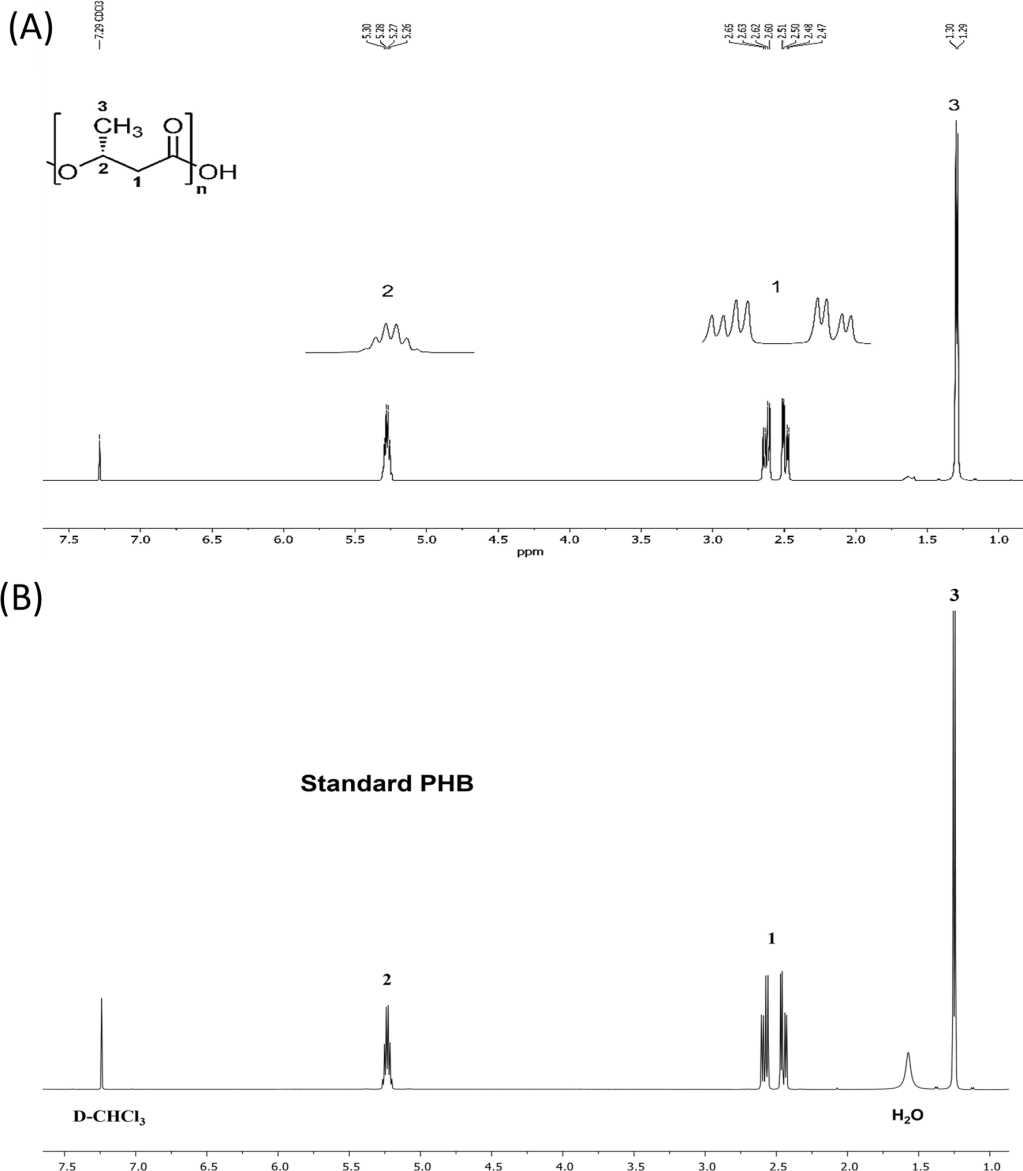
500 MHz ^1^H NMR spectrum of **A** P(3HB) produced by *Cupriavidus* sp. CB15 and **B** P(3HB) standard (Sigma-Aldrich, USA)

The chemical structure of the extracted polymer was investigated by ^1^H NMR. From the spectrum in Fig. 8, the extracted polymer was confirmed as P(3HB) homopolymer, which corresponded to the spectrum of P(3HB) standard and a previous report [53]. The methyl proton of 3HB was assigned at δ1.29–1.30 ppm. The methylene proton was assigned at the regions of δ2.47–2.65 ppm. The methine proton was around δ5.26–5.30 ppm.

## Conclusion

This study was aimed to isolate a novel thermotolerant bacterium that is capable of synthesizing PHA and optimize its PHA production. Among all the isolates, the strain CB15 gave the highest PHA production using glycerol as a sole carbon source. This isolate was a gram-negative bacterium, which was classified as a member of the genus *Cupriavidus*. Nutritional and physical parameters were optimized using OFAT followed by RSM with rotatable CCD. The optimum conditions for the growth and PHA production were 1% (w/v) glycerol, 0.78 g/L NH_4_Cl, incubation at 45 °C, and shaking speed at 175 rpm for 72 h with PHA concentration of 2.01 g/L and DCW of 2.87 g/L. The extracted PHA was characterized by ^1^H NMR confirming its structure was P(3HB). According to the results in this study, *Cupriavidus* sp. CB15 is a promising candidate for PHA production. Further scale-up studies using the optimized conditions will be carried out under controlled condition on a bioreactor scale. Fed-batch cultivation will be applied to enhance PHA productivity. In addition, the ability of *Cupriavidus* sp. CB15 to synthesize copolymer such as P(3HB-*co*-3HV) by supplementation of appropriate precursor compounds will be observed.

## Supplementary Information

**Additional file 1: Table S1 **Basic properties of compost samplings.

## Data Availability

The datasets generated and analyzed during the current study are available from the corresponding author on reasonable request.
