# Supplementary material for: Characterization of newly isolated thermotolerant bacterium Cupriavidus sp. CB15 from composting and its ability to produce polyhydroxyalkanoate from glycerol
Source: Microb Cell Fact. 2023 Apr 12;22:68. doi: 10.1186/s12934-023-02059-5 (PMC10091600; doi:10.1186/s12934-023-02059-5)
Supplement: Supplementary file 1 — Additional file 1: Table S1 Basic properties of compost samplings. [file 12934_2023_2059_MOESM1_ESM.docx]

**Additional file 1 : Table S1** Basic properties of compost samplings

|  | Samples | | | | |
| --- | --- | --- | --- | --- | --- |
| Parameters | **Corncob compost 1** | **Corncob compost 2** | **Coconut coir compost** | **Coffee pulp compost** | **Leave compost** |
| pH | 7.81 | 7.42 | 6.46 | 7.34 | 7.86 |
| %Ash | 3.38 | 46.04 | 4.97 | 26.57 | 32.49 |
| %Moisture | 62.96 | 9.01 | 84.13 | 51.01 | 15.96 |
| %Dry material | 37.04 | 90.99 | 15.87 | 48.99 | 84.04 |
| %Carbon | 53.68 | 29.97 | 52.78 | 40.80 | 37.50 |
| %Nitrogen | 0.09 | 1.52 | 0.10 | 0.26 | 0.42 |

Means of duplicate experiments are shown.
